# Eating Disorder Examination Questionnaire (EDE-Q-13): expanding on the short form

**DOI:** 10.1186/s40337-021-00403-x

**Published:** 2021-04-29

**Authors:** Lilac Lev-Ari, Rachel Bachner-Melman, Ada H. Zohar

**Affiliations:** 1 Clinical Psychology Graduate Program, Ruppin Academic Center, Emek Hefer, Israel; 2 Lior Zfaty Suicide and Mental Pain Research Center, Emek Hefer, Israel; 3 School of Social Work, Hebrew University of Jerusalem, Jerusalem, Israel

**Keywords:** EDE-Q, Eating disorders, Assessment, EDE-Q-7, EDE-Q-13

## Abstract

**Objective:**

The Eating Disorders Examination–Questionnaire (EDE-Q) is widely used but time-consuming to complete. In recent years, the advantages and disadvantages of several brief versions have therefore been investigated. A seven-item scale (EDE-Q-7) has excellent psychometric properties but excludes items on bingeing and purging. This study aimed to evaluate a thirteen-item scale (EDE-Q-13) including items on bingeing and purging.

**Method:**

Participants were 1160 (188 [11.4%] males) community volunteers of mean age 28.79 ± 9.92. They completed the full EDE-Q in Hebrew, as well as measures of positive body experience, social and emotional connection, life satisfaction, positive and negative affect and positive eating. The six EDE-Q items about bingeing and purging, recoded to correspond to the response categories of the other EDE-Q questions, were added to the EDE-Q-7, resulting in the EDE-Q-13.

**Results:**

Confirmatory factor analysis confirmed the hypothesized EDE-Q-13 structure, including the bingeing and purging subscales. Strong positive correlations were found between the EDE-Q-13 and the original EDE-Q scores. The EDE-Q-13 showed convergent validity with related measures.

**Conclusions:**

The EDE-Q-13 in Hebrew is a brief version of the EDE-Q that includes bingeing and purging subscales and has satisfactory psychometric properties. Its use in clinical and research contexts is encouraged.

## Plain English summary

The Eating Disorders Examination–Questionnaire (EDE-Q) is a widely used questionnaire that assesses eating disorder symptoms, however its 28 items take time to complete. In this study we examined a 13-item version of the EDE-Q, consisting of a seven-item version shown to have good properties and six EDE-Q items assessing binge eating and purging. The full EDE-Q and measures of positive body experience, social and emotional connection, life satisfaction, positive and negative affect and positive eating were completed online by 1160 community volunteers (11.4% males) between 18 and 76 years of age. The expected structure of the EDE-Q-13 was confirmed. EDE-Q-13 and the original EDE-Q scores were highly correlated, and the EDE-Q-13 was associated with questionnaires that are associated with the EDE-Q. The EDE-Q-13 is a brief version of the EDE-Q that includes bingeing and purging subscales. It can be used to estimate the presence of eating disorder symptoms in community samples.

## Introduction

The EDE-Q has been in use for over quarter of a century [1] and has been translated into many languages, including Hebrew [2]. This widely used self-report questionnaire discriminates between disordered eating and eating disorders in screening community samples [3], in primary care [4] and supports the clinical diagnosis of eating disorders [5].

The four subscales of the EDE-Q were originally determined in a clinical interview [6] (as Restraint, and Eating-, Weight- and Shape- Concern. Subsequently, some confirmatory factor analyses have found that weight and shape concern were better considered as a single factor [2, 7, 8], so that a three-factor structure was recommended. Other studies have suggested alternate four-factor structures [9].

Because of the usefulness of the EDE-Q clinically, epidemiologically, and in basic research, there have been several attempts to derive a short form of the questionnaire. Grilo et al. [8] administered the EDE-Q to a sample of undergraduate students in the United States, and found, using confirmatory factor analysis, a 3-factor solution: Dietary Restraint, Shape/Weight overvaluation, and Body Dissatisfaction. In their analyses, they found that a short seven-item form not only reproduced this hypothesized three-factor solution, but that it showed higher convergent validity than did the longer versions of the EDE-Q they considered. However, the seven items selected did not include any that measured bingeing or purging.

A 12-item version of the EDE-Q was suggested by Gideon et al. [10], who derived the items in a careful two-stage process using participants with and without clinical eating disorders. They changed the response scale of the items to a four-point scale to improve the response distribution and excluded less informative items. The 12 items selected included two pertaining to bingeing; the other items related to restriction and body and weight concerns. This 12-item scale was shown to have excellent internal reliability, test-retest reliability, and to distinguish between participants with and without eating disorders. The time frame for reference was the previous week. Gideon et al. [10] found the 12-items were best described as a single factor and suggested using their 12-item EDE-Q as a user-friendly, weekly assessment of treatment efficacy in eating disorder facilities. The good psychometric properties of the 12-item version were replicated in a Mandarin translation administered to Chinese university students [11]. Further analysis of the Gideon et al. [10] data by another research group [12] calculated a cut-off score for optimal sensitivity and specificity for the 12-item version, increasing its usefulness as a screening tool.

Careful systematic work on large samples of British adult females and males in the community resulted in an 18-item version of the EDE-Q for females, and a 16-item version for males. The 18-item version was subsequently validated for females, with a three-factor solution: Shape and Weight Concern, Preoccupation and Eating Concern, and Restriction [7]. The 16-item version for males produced a similar item structure, which required further confirmation.

Machado et al. [13] recruited individuals receiving treatment at two eating disorder clinics and a large control group of community volunteers to compare several short versions of the EDE-Q to the original Fairburn and Beglin [1] 28-item questionnaire. While specificity and sensitivity of the Carey et al. [7] 18-item and the Kliem et al. [14] eight-item versions were adequate, only the Grilo et al. [8] seven-item version retained the three-factor structure found in the original 28-item EDE-Q. Based on these results, Machado et al. [13] recommended the use of the seven-item version of the EDE-Q together with the final six bingeing and purging items of the original 28-item scale. However, the six bingeing and purging items (e.g. “in the past 28 days how many times have you eaten what other people would regard as an unusually large amount of food [given the circumstances]?”) require open numerical responses questions allowing answers between 0 and infinity, whereas the response categories for the other items are grouped, for example “no days”, “1–5 days”, 6–12 days”. People with EDs tend to inflate the number of their bingeing and purging episodes, resulting in inadequate reliability for these subscales that are therefore generally excluded from analyses [15]. An advantage of including the bingeing and purging items (with adapted response scale) is that they focus on behaviors, and therefore complement the items that relate to weight and shape (over)concern.

*The main thrust of this study was to produce a short, user-friendly version of the EDEQ that would not only retain the excellent psychometric qualities of some of the other short versions (*e.g. *the 12-item version), but also the three-factor structure of the original 28-item EDEQ, enabling specific concepts to be measured. W*e examine the use of a 13-item version of the EDE-Q that included Grilo et al.’s [8] seven-item version and the six items about bingeing and purging recoded so that their response categories correspond to those of the other items. The 13 items were hypothesized to conform to a five-factor solution: The original three factors of the Grilo et al. [8] seven-item version i.e. Dietary Restraint, Shape and Weight Over-evaluation, Body Dissatisfaction, as well as a Bingeing and a Purging factor, missing from the other short versions of the EDE-Q. We chose to use a community sample, because this enabled participants (both women and men) of different ages to participate, and because a short, parsimonious questionnaire seems particularly suitable for use in the community. We examined convergent validity by observing the pattern of correlations between EDE-Q-13 scores with measures of concepts that are related to ED symptomatology. These concepts include positive body experiences or body image, and enjoyment from (positive) eating, which can be expected to be negatively associated with ED symptoms. We also chose to include emotional, affective and social variables that have been shown to be associated with recovery from ED, namely positive and negative affect, life satisfaction and social and emotional connection [16].

We hypothesized that:

1. The EDE-Q-13 would demonstrate good construct structure for a five-factor model: Eating restraint, Shape and Weight Over-evaluation, Body Dissatisfaction, Bingeing and Purging (using confirmatory factor analysis [CFA]).
2. Total and subscale scores of the EDE-Q-13 would correlate strongly with the original EDE-Q total and subscale scores.
3. EDE-Q-13 total scores (and the original EDE-Q scores) would correlate negatively with measures of positive body experiences, positive affect, positive eating, life satisfaction and social and emotional connection and positively with negative affect.
4. EDE-Q-13 total scores would yield a pattern of correlations similar to that yielded by the 28-item EDE-Q total scores.

## Method

### Participants

A total of 1160 (188; 11.4% males) Israeli community volunteers between 18 and 76 years of age (M = 28.79, SD = 9.92) registered online to participate in the study. Half of the participants were recruited via the social media and the other half via an introductory psychology course (in a college in the middle of Israel), for which they received class credit. Two thirds (65.9%) of the participants were single, 368 (31.7%) were married and 54 (4.7%) were divorced or reported “other” status. 89.8% were Jewish, 8.7% were Muslim, .7% were Christian and the rest (.8%) were ‘other’ or did not wish to reply. They had 0–11 children (M = 1.16, SD = 1.65) and a mean of 13.98 years of schooling (SD = 2.23). Their body mass index (BMI) ranged between 16.31 and 53.15 (M = 23.46, SD = 5.11).

### Measures

#### Eating disorder symptoms

##### Ede-q

Eating disorder symptoms were assessed using the original version of the Eating Disorders Examination – Questionnaire [1]. The EDE-Q was translated into Hebrew with permission [2], using a process of translation, independent back translation and revision. It contains 28 items assessing core eating disorder symptoms related cognitions, and includes four subscales, each containing five to eight items. The instructions for answering the questionnaire are “In the past 28 days,”: 1) Dietary Restraint (DR) e.g. “How often have you been deliberately trying to limit the amount of food you eat to influence your shape or weight [whether or not you have succeeded]?”; 2) Eating Concern (EC) e.g. “How concerned have you been about other people seeing you eat?”; 3) Weight Concern (WC) e.g. “How often have you had a definite fear that you might gain weight?”; and 4) Shape Concern (SC) e.g. “How often have you had a definite desire to have a totally flat stomach?”. A global score averaging the subscales is also used. The responses to 22 items are rated using a seven-point forced-choice format from 0 to 6. For some questions the answers are 0 ‘0 days’, 1 ‘1–5 days’, 2 ‘6–12 days’, 3 ‘13–15 days’, 4 ‘16–22 days’, 5 ‘23–27 days’ and 6’every day’. For some of the questions, the answers range from 0 ‘never’ to 6 ‘always’; and for some questions from 0 ‘not at all’ to 6 ‘very much’. Higher scores reflect greater symptom severity. The remaining six items about the frequency of binge eating and compensatory behaviors require open, numerical responses, are used for diagnostic purposes and are generally excluded from factor analyses. A cut-off of four (for subscales and the global score) indicates risk for a clinical eating disorder, for both men and women [17]. Zohar et al. [2] assessed 292 community volunteers and found sound psychometric properties for the Hebrew translation but recommended combining WC and SC into one subscale. In the current study, the internal reliability for the total score and all subscales was acceptable (Cronbach’s alpha > .78).

##### EDE-Q-13

Our proposed version of the EDE-Q contains seven items from the original questionnaire as suggested by Machado et al. [13] that were pulled from the complete EDE-Q. These items are the original items 1, 3 and 4 that assess DR, items 22 and 23 that assess WC and SC (Shape and Weight Over-evaluation [SWO], as in Machado et al. [13], and items 25 and 26 that measure BD. For psychometric as well as content purposes, we unified response formats for all items. The questionnaire opens with a phrase relevant to all questions, ‘On how many of the past 28 days ......’, and the 12 questions that follow ask about specific thoughts or behaviors. Response options are six frequency categories: 1–5 (score of 1); 6–12 (score of 2); 13–15 (score of 3); 16–22 (score of 4); 23–27 (score of 5); and every day (score of 6). The six open-ended Bingeing (e.g. “You felt a loss of control over your food as you were eating”) and Purging (e.g. “You made yourself vomit in order to control your weight”) items that appear at the end of the 28-item EDE-Q were recoded and reformatted with the same frequency response categories and included in the scoring of the EDE-Q-13, following the general recommendation of Machado et al. [13]. It should be noted, however, that although the adaptation of these items into a Likert-type response format was not suggested by Machado et al. [13], we initiated this step so that scoring would be uniform. The EDE-Q-13 appears in Appendix.

#### Positive body experiences

Positive body experiences were measured by the Dresden Body Image Questionnaire-35 (DKB-35) [18, 19];. The DKB-35 is a 35-item scale presenting a positive and comprehensive conceptualization of body image, originally validated in German. In a community sample of 349 men and women, the German questionnaire was shown to be reliable and valid with internal consistency of the subscales ranging between 0.76 and 0.91. The scale showed good construct validity and stability over 7 days [18]. It was translated into Hebrew and English following the star paradigm with permission from the authors [2, 20]. The Hebrew version used in this study has shown good reliability and validity [2]. Its five subscales, rated between 1 (“not at all true for me”) and 5 (“very true for me”), are: 1) Vitality e.g. “I am physically fit”; 2) Body Narcissism (BN) e.g. “I find it pleasant and stimulating when somebody looks at me attentively”; 3) Sexual Fulfillment (SF) e.g. “I feel my body pleasantly and intensely in sexuality”; 4) Body Acceptance (BA) e.g. “I am satisfied with how I look”; and 5) Physical Contact (PC) e.g. “Physical contact is important for me to express closeness.” The subscales displayed excellent reliability, with Cronbach’s alphas ranging between 0.80 and 0.90.

#### Social and emotional connection

Social and emotional connection was assessed using the seven-item Social and Emotional Connection (SEC) subscale of the Eating Disorders Recovery Questionnaire (EDRQ) [21]. The EDRQ is a 28-item, psychometrically sound questionnaire assessing recovery from an eating disorder. Its other subscales are Physical Health, Lack of Symptoms and Body Acceptance. Sample items for this SEC subscale are “I am in touch with my own feelings” and “I am able to express my emotions in words”. The original scale was written in Hebrew, and alpha’s Cronbach was 0.92 [21]. Responses are noted on a seven-point Likert scale between 0 (I do not agree at all) and 6 (I completely agree), with higher scores reflecting fewer problems with emotional and social connection. The alpha Cronbach of the SEC subscale of the EDRQ was 0.92.

#### Life satisfaction

Life Satisfaction was assessed using the Satisfaction with Life Scale (SWLS) [22]. The SWLS contains five items that cognitively appraise the respondents’ life in general. The SWLS is a common measure of well-being and has good psychometric properties [22]. Items are scored between 1 (“strongly disagree”) and 7 (“strongly agree”), with high scores indicating greater life satisfaction. A Hebrew version previously used in research was administered in this study [23]. The alpha Cronbach in this study was 0.89.

#### Positive and negative affect

Positive and negative affect were assessed via the Positive and Negative Affect Schedule – Short Form (PANAS-SF) [24]. The PANAS-SF is a ten-item questionnaire with five items about positive affect (PANAS-SF-Pos) and five about negative affect (PANAS-SF-Neg). Respondents were asked to report the strength with which they usually feel emotions such as excitement or anger on a five-point Likert scale between 1 (“hardly at all”) to 5 (“very strongly”). The PANAS-SF has been shown to have good validity and reliability in various cultures [24]. A Hebrew translation previously used in research [25] was administered in this study. The alpha Cronbach in this study was 0.79 for positive affect and 0.83 for negative affect.

#### Positive eating

Positive eating was reported by completing the Positive Eating Scale (PES) [26], an eight-item questionnaire that asks about enjoyment of eating. It has two subscales that assess Satisfaction with Eating (e.g. “I am relaxed about eating”) and Pleasure when Eating (e.g. “Eating is fun for me”). The PES was validated and has been shown to have good psychometric properties and the same structure in a large longitudinal community sample (*n* = 772) from Germany, India and the US, with alpha Cronbach 0.87 [26]. Six-month test-retest reliability was 0.67 [26]. Items are scored on a five-point Likert scale between 1 (“I strongly disagree”) and 4 (“I strongly agree”). A Hebrew translation (used in [26]) was used in this study, and the alpha Cronbach was 0.93.

### Procedure

The study received approval from the Institutional Internal Review Board. Participants were sent a link to the questionnaires, which they completed online. A full explanation about the study was provided on the first screen, and informed consent was provided. Participants reported on demographic information, height and weight, before completing the questionnaires. All participants completed the EDE-Q (original version) and the DKB-35 and a subset of 960 participants also completed the PANAS-SF, PES, SWLS, and SEC. The EDE-Q was then completed twice, once using the original format and once using the EDE-Q-13 format. The Bingeing and Purging questions that required open numerical responses in the original format were recoded and rescored in accordance with the other items for the EDE-Q-13. On the last screen, contact details of the researchers were provided and participants were encouraged to send them questions, comments or difficulties.

### Data analysis

AMOS 23.0 was used for the CFA. To test for convergent validity, Pearson correlations were calculated between EDE-Q-13 total scores and positive body experiences (DKB-35), positive eating (PES), positive and negative affect (PANAS-SF), satisfaction with life (SWLS) and social and emotional connection (SEC). Analyses were conducted using the Statistical Package for the Social Sciences (SPSS, version 23).

## Results

- Hypothesis 1: *The EDE-Q-13 would demonstrate good construct structure (using CFA)*.

### CFA of EDE-Q-13 (***N*** = 1160)

**Fig. 1:**
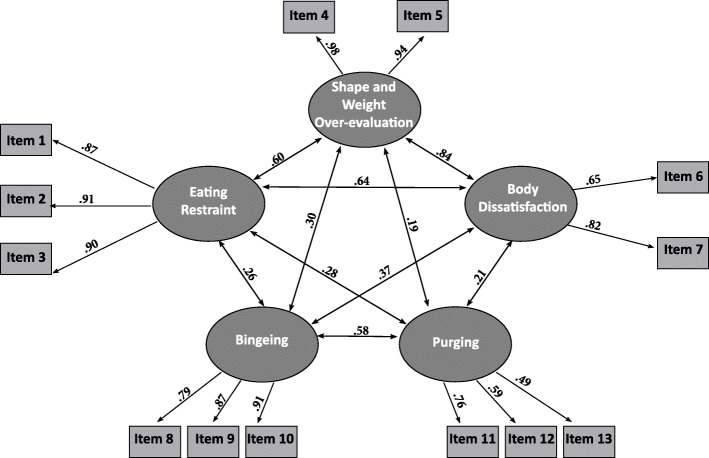
CFA of the five-factor EDE-Q-13 model. Note: Ellipses indicate latent variables. Rectangles indicate observed variables. Arrows between latent variables indicate significant correlations between latent variables. Correlations between latent and observed variables were significant at *p* < .001

CFA was used to test the hypothesized structure of the EDE-Q-13. This analysis examines the consistency of constructs as they are conceptualized theoretically or empirically. The following values were chosen for acceptance of the hypothesized structure: Comparative Fit Index (CFI) > .90 [27], root mean square error of approximation (RMSEA) < .08 [28] and SRMR<.08 (see Fig. 1). The model showed good fit (χ^2^_(55)_ = 282.63; *p* < .001; CFI = .98, RMSEA = .05; SRMR = .04). Cronbach’s alphas for the EDE-Q-13 subscales were .99 for SWO, .89 for BD, .92 for ER, .89 for Bingeing and .63 for Purging.

- Hypothesis 2: *Total and subscale scores of the EDE-Q-13 would correlate strongly with the original EDE-Q total and subscale scores (n = 1160)*

Pearson correlations between the EDE-Q-13 subscales and the original EDE-Q subscales are presented in Table 1. All correlations were significant at *p* < .001 and ranged between .29 and .95. The correlation between EDE-Q-13 total scores and the original EDE-Q total score was .92.

**Table 1:** Correlations between the EDE-Q-13 and the original EDE-Q total and subscales

Original EDE-Q EDE-Q-13	Eating Restraint	Eating Concerns	Shape and Weight Concerns	EDE-Q total
Eating Restraint	.95	.58	.66	.82
Shape and Weight Overevaluation	.61	.65	.86	.78
Body Dissatisfaction	.59	.60	.89	.77
Bingeing	.30	.50	.35	.41
Purging	.30	.28	.22	.29
EDE-Q-13 total	.86	.75	.88	.92

Note: All correlations were significant at the *p* < .001 (2-tailed). Pearson correlations between .0–.30 (positive or negative) are considered to be of low strength, between .30–.60 of medium strength and above .60 as strong

Pearson inter-correlations between the EDE-Q-13 subscales are presented in Table 2. All correlations were significant at *p* < .001 and ranged between .15 and .83. The mean for Purging was lowest (1.43) and all other means ranged between 3.41–3.93.

- Hypotheses 3 and 4: *EDE-Q-13 total scores (and the original EDE-Q scores) would correlate negatively with measures of positive body experiences (DKB-35), positive affect (PANAS-SF-Pos), positive eating (PES), life satisfaction (SWLS) and social and emotional connection (SEC) and positively with negative affect (PANAS-SF-Neg). EDE-Q-13 total scores would yield a pattern of correlations similar to that yielded by the 28-item EDE-Q total scores.*

**Table 2:** Intercorrelations between the EDE-Q-13 subscales (*n* = 1160)

	Eating Restraint	Shape and Weight Overevaluation	Body Dissatisfaction	Bingeing	Purging	EDE-Q-13 total
Eating Restraint		.57	.54	.24	.26	.83
Shape and Weight Overevaluation			.72	.28	.16	.81
Body Dissatisfaction				.30	.15	.80
Bingeing					.42	.53
Purging						.42
Mean (SD)	3.50 (2.19)	3.72 (2.11)	3.93 (2.00)	3.41 (6.47)	1.43 (3.15)	3.11 (2.35)

Note: All correlations were significant at the *p* < .001 (2-tailed). Pearson correlations between .0–.30 (positive or negative) are considered to be of low strength, between .30–.60 of medium strength and above .60 as strong

Pearson correlations between the EDE-Q-13 and the original EDE-Q total scores and the *DKB-35, PANAS-SF, PES, SWLS and SEC* are presented in Table 3. All correlations were significant at *p* < .001 and ranged between −.09 and .69. The correlations of the EDE-Q-13 total score and the original EDE-Q total score with other variables assessing body acceptance and psychological well-being were similar. These findings further our understanding of the validity of the EDE-Q-13, which showed high convergent validity with body acceptance, negative affect and positive eating and divergent validity with vitality, body narcissism, physical contact, positive eating and psychological wellbeing.

**Table 3:** Correlations between a. total EDE-Q-13 and 28-item EDE-Q scores and b. DKB-35, PANAS-SF, PES, SWLS and SEC scores (*n* = 960)

		DKB-35				PANAS-SF				
	Vitality	BA	BN	SF	PC	Pos	Neg	PES	SWLS	SEC
EDE-Q-13 total	−.31	−.51	−.09	−.28	−.15	−.13	.45	−.53	−.29	−.35
Original EDE-Q total	−.37	−.69	−.14	−.37	−.20	−.13	.50	−.63	−.34	−.37

Note: All correlations were significant at *p* < .001 (2-tailed). *DKB-35* Dresden Body Image Questionnaire-35, *PANAS-SF* Positive And Negative Affect Scale – Short Form, *Vitality* DKB-35 Vitality subscale, *BA* DKB-35 Body Acceptance subscale, *BN* DKB-35 Body Narcissism subscale, *SF* DKB-35 Sexual Fulfillment subscale, *PC* DKB-35 Physical Contact subscale, *Pos* PANAS-SF Positive subscale, *Neg* PANAS-SF Negative subscale, *PES* Positive Eating Scale, *SWLS* Satisfaction with Life, *SEC* Social and Emotional Connection subscale of the Eating Disorder Recovery Questionnaire

Pearson correlations between .0–.30 (positive or negative) are considered to be of low strength, between .30–.60 of medium strength and above .60 as strong

To verify that bingeing and purging behaviors are valid indications of the severity of eating pathology, we compared EDE-Q-13 scores, excluding the Bingeing and Purging items respectively, for participants who reported some versus no bingeing and participants who reported some versus no purging. Over half (53%) of the participants reported no purging and 37% reported no bingeing. Two one-sided t-tests showed that participants who reported no bingeing had lower total scores on the remaining seven items of the EDE-Q-13 (mean = 3.27, SD = 1.77) than those who reported bingeing (mean = 3.96, SD = 1.80; t = − 7.49, *p* < .001), and that participants who reported no purging had lower total scores on the seven remaining items of the EDE-Q-13 (mean = 3.56, SD = 1.79) than those who reported purging (mean = 3.84, SD = 1.84; t = − 3.14, *p* = .002).

## Discussion

The purpose of this study was to compare the Hebrew version of the 13-item EDE-Q-13 with that the Hebrew translation of the complete 28-item EDE-Q. The responses to the bingeing and purging items of the original questionnaire were restructured and included in the scoring of the short version. The structure of the scales was compared using CFA and the pattern of correlations between the total and subscale scores of both questionnaires was observed, as well as the pattern of correlations between EDE-Q-13 and EDE-Q total scores respectively with several scales measuring related variables.

Our results supported a five-factor model for the EDE-Q-13, with subscale scores for Eating Restraint, Body Dissatisfaction, Shape and Weight Over-evaluation, Bingeing and Purging. This factor structure found for the EDE-Q-13 replicated the factor structure of the EDE-Q7 presented in Machado et al. [13] and added Bingeing and Purging subscales. It also replicated two of the four original factors in the 28-item EDE-Q: Restraint subscale with the Weight and Shape Concern items combined into a single factor (Shape and Weight Overevaluation) as in many previous studies [2, 29]. A major disadvantage of the full EDE-Q to date is that the open-ended structure of the response categories of the Bingeing and Purging items has prevented them from being included in scoring and data analyses. The recoding of these items and the inclusion of Bingeing and Purging subscales in the EDE-Q-13 score is therefore a major advantage of this short version of the questionnaire. Participants who scored above 1 on the Bingeing or Purging subscales scored higher on the EDE-Q-13 total scores excluding these two subscales, supporting the importance of these additional items.

Another major advantage of the EDE-Q-13 is that it is short, user-friendly and parsimonious. Its total and subscale scores correlated strongly with those of the 28-item EDE-Q, so that significant information does not seem to be missed when it is used in lieu of the longer version, and it preserves the central features of the EDE-Q. The correlations of the Purging subscale, and to a lesser extent the Bingeing subscale, with the other subscales and with the original EDE-Q total tended to be low. This could be explained by the low levels of purging (and bingeing) observed in our community sample and the resulting restricted range of scores. Correlations and intercorrelations should therefore be examined in clinical samples. The EDE-Q-13 also showed convergent validity. Participants who reported higher levels of eating disorder symptoms tended to have significantly lower levels of positive body experiences, positive affect, positive eating, life satisfaction and social and emotional connection to others, and significantly higher levels of negative affect. Although the strength of the correlations between EDE-Q-13 scores and body satisfaction, affect, positive eating, psychological well-being and personal contact could be interpreted as small to medium, they were in line with those between the long version of the EDE-Q and the other measures.

Our study has limitations. First, the version of the EDE-Q-13 used in this study was in Hebrew, so its psychometric properties should be verified in other languages. Since it was administered in Hebrew, it included predominantly Jewish Israelis; an Arabic version would be helpful for assessing Israeli Arabs. Second, this study was conducted with a community sample of predominantly female, single, educated community sample and may therefore not be generalizable to other populations. This may also be a reason for the somewhat low reliability of the Purging subscale. Third, although the use of the Likert format for the binge/purge items allows researchers and clinicians to incorporate behavioral frequency information within a continuous subscale or global scale score, it also obscures the actual frequency of binge eating/purging, such that it no longer becomes possible to determine whether participants reported “clinical” levels of these behaviors (i.e., 4x/month). It is also unclear whether adding scores for bingeing and purging behaviors may result in some respondents with these behaviors receiving higher scores on the total scale that may or may not be warranted. Further studies should investigate the validity of the EDE-Q-13 in clinical settings, its ability to accurately distinguish between cases and controls and its sensitivity to change.

The EDE-Q is widely used, but reporting on the full version is time-consuming, and presents significant participant burden, which may deter some respondents from completing the entire questionnaire. Researchers wishing to use a short version of the questionnaire have tried to decide which version is most useful [13] Although the shortest version suggested had only seven items and excellent psychometric properties, it omitted to ask about bingeing and purging. Thus, the EDE-Q-13 builds on the seven-item version but adds bingeing and purging items, important in assessing ED symptomatology. The EDE-Q-13 makes self-report less burdensome in two distinct ways: it is more than 50% shorter than the original version, and it has a unified response scale. Future research should try and validate this version of the EDE-Q in other languages and in clinical settings.

## Conclusions

We found that the EDE-Q-13 was reliable and showed convergent validity. It is possible to use this short and user-friendly self-report to estimate the presence of eating disorder symptoms in community samples for research and clinical purposes.

## Data Availability

All data and materials are available upon request.

## Appendix

### EDE-Q-13

Instructions: The following questions adress the past 4 weeks (28 days) only. Please read each question carefully. Please answer all the questions and choose one answer for each question. Thank you.

Remember that the questions only refer to the past 4 weeks (28 days) only.

**Table:**

			0	1	2	3	4	5	6
EDE-Q	EDE-Q-13	On how many of the past 28 days ......	0	1–5	6–12	13–15	16–22	23–27	Every day
1.	1.	have you been deliberately trying to limit the amount of food you eat to influence your shape or weight (whether or not you have succeeded)?							
3.	2.	have you tried to exclude from your diet any foods that you like in order to influence your shape or weight (whether or not you have succeeded)?							
4.	3.	have you tried to follow definite rules regarding your eating (for example, a calorie limit) in order to influence your shape or weight (whether or not you have succeeded)?							
22.	4.	has your weight influenced how you think about (judge) yourself as a person?							
23.	5.	has your shape influenced how you think about (judge) yourself as a person?							
25.	6.	have you been dissatisfied your weight?							
26.	7.	have you been dissatisfied your shape?							
13.	8.	have you eaten what other people would regard as an unusually large amount of food (given the circumstances)?							
14.	9.	did you have a sense of having lost control over your eating (at the time that you were eating)?							
15.	10.	have such episodes of overeating occurred (i.e. you have eaten an unusually large amount of food and have had a sense of loss of control at the time)?							
16.	11.	have you made yourself sick (vomit) as a means of controlling your shape or weight?							
17.	12.	have you taken laxatives as a means of controlling your shape or weight?							
18.	13.	have you exercised in a “driven” or “compulsive” way as a means of controlling your weight, shape or amount of fat or to burn off calories?							

Questions 1–3: Restricted Eating; 4–5 Shape and Weight Over-evaluation; 6–7 Body Dissatisfaction; 13–15 Bingeing; 16–18 Purging.

## Abbreviations

EDE-Q: Eating Disorders Examination–Questionnaire
EDE-Q-7: Eating Disorders Examination–Questionnaire – seven items
EDE-Q-13: Eating Disorders Examination–Questionnaire – thirteen items
DR: Dietary Restraint
EC: Eating Concern
WC: Weight Concern
SC: Shape Concern
DKB-35: The Dresden Body Image Questionnaire-35
BN: Body Narcissism
SF: Sexual Fulfillment
BA: Body Acceptance
PC: Physical Contact
SEC: Social and Emotional Connection
EDRQ: The Eating Disorders Recovery Questionnaire
SWLS: The Satisfaction with Life Scale
PANAS-SF: The Positive and Negative Affect Schedule – Short Form
PANAS-SF-Pos: Positive affect
PANAS-SF-Neg: Negative affect
PES: The Positive Eating Scale
CFA: Confirmatory factor analysis
CFI: Comparative Fit Index
RMSEA: Root mean square error of approximation

## References

[1] Fairburn CG, Beglin SJ (1994). Assessment of eating disorders: interview or self-report questionnaire?. Int J Eat Disord..

[2] Zohar AH, Lev-Ari L, Bachner-Melman R (2017). The EDE-Q in Hebrew: structural and convergent/divergent validity in a population sample. Isr J Psychiatry Relat Sci.

[3] Mond JM, Hay PJ, Rodgers B, Owen C (2006). Eating disorder examination questionnaire (EDE-Q): norms for young adult women. Behav Res Ther.

[4] Mond JM, Myers TC, Crosby RD, Hay PJ, Rodgers B, Morgan JF, Hubert Lacey J, Mitchell JE (2008). Screening for eating disorders in primary care: EDE-Q versus SCOFF. Behav Res Ther.

[5] Schaefer LM, Smith KE, Leonard R, Wetterneck C, Smith B, Farrell N, Riemann BC, Frederick DA, Schaumberg K, Klump KL, Anderson DA, Thompson JK (2018). Identifying a male clinical cutoff on the eating disorder examination-questionnaire (EDE-Q). Int J Eat Disord..

[6] Cooper Z, Fairburn C (1987). The eating disorder examination: a semi-structured interview for the assessment of the specific psychopathology of eating disorders. Int J Eat Disord..

[7] Carey M, Kupeli N, Knight R, Troop NA, Jenkinson PM, Preston C (2019). Eating disorder examination questionnaire (EDE-Q): norms and psychometric properties in UK females and males. Psychol Assess.

[8] Grilo CM, Reas DL, Hopwood CJ, Crosby RD (2015). Factor structure and construct validity of the eating disorder examination-questionnaire in college students: further support for a modified brief version. Int J Eat Disord.

[9] Friborg O, Reas DL, Rosenvinge JH, Rø Ø (2013). Core pathology of eating disorders as measured by the eating disorder examination questionnaire (EDE-Q): the predictive role of a nested general (g) and primary factors. Int J Methods Psychiatr Res.

[10] Gideon N, Hawkes N, Mond J, Saunders R, Tchanturia K, Serpell L (2016). Development and psychometric validation of the EDE-QS, a 12 item short form of the eating disorder examination questionnaire (EDE-Q). PLoS One.

[11] He J, Sun S, Fan X (2020). Validation of the 12-item short form of the eating disorder examination questionnaire in the Chinese context: confirmatory factor analysis and Rasch analysis. Eat Weight Disord.

[12] Prnjak K, Mitchison D, Griffiths S, Mond J, Gideon N, Serpell L, Hay P (2020). Further development of the 12-item EDE-QS: identifying a cut-off for screening purposes. BMC Psychiatry.

[13] Machado PP, Grilo CM, Rodrigues TF, Vaz AR, Crosby RD (2020). Eating disorder examination–questionnaire short forms: a comparison. Int J Eat Disord..

[14] Kliem S, Mößle T, Zenger M, Strauß B, Brähler E, Hilbert A (2016). The Eating Disorder Examination-Questionnaire 8: A brief measure of eating disorder psychopathology (EDE-Q8). Int J Eat Disord.

[15] Goldfein JA, Devlin MJ, Kamenetz C (2005). Eating disorder examination-questionnaire with and without instruction to assess binge eating in patients with binge eating disorder. Int J Eat Disord..

[16] Bachner-Melman R, Lev-Ari L, Zohar AH, Lev SL (2018). Can recovery from an eating disorder be measured? Toward a standardized questionnaire. Front Psychol.

[17] Luce KH, Crowther JH, Pole M (2008). Eating disorder examination questionnaire (EDE-Q): norms for undergraduate women. Int J Eat Disord..

[18] Matthes J, Franke GH, Jäger S (2012). Psychometrische Prüfung des Dresdner Körperbildfragebogens (DKB-35) in einer nicht-klinischen Stichprobe. Z Med Psychol.

[19] Pöhlmann K, Roth M, Braehler E, Joraschky P (2013). The Dresden body image inventory (DKB-35): validity in a clinical sample. Psychother Psychosom Medizinische Psychol.

[20] Lev-Ari L, Zohar AH, Bachner-Melman R. Enjoying your body: The psychometric properties of an English version of the Dresden Body Image Questionnaire. Aust J Psychol. 2020;72(4). 10.1111/ajpy.12284.

[21] Bachner-Melman R, Lev-Ari L, Zohar AH, Linketsky M. The eating disorders recovery questionnaire: psychometric properties and validity. Eat Weight Disord. 2021:1–11 10.1007/s40519-021-01139-y. 10.1007/s40519-021-01139-y 33582972

[22] Diener ED, Emmons RA, Larsen RJ, Griffin S (1985). The satisfaction with life scale. J Pers Assess.

[23] Shmotkin D, Lomranz J (1998). Subjective well-being among holocaust survivors: an examination of overlooked differentiations. J Pers Soc Psychol.

[24] Thompson ER (2007). Development and validation of an internationally reliable short-form of the positive and negative affect schedule (PANAS). J Cross-Cult Psychol.

[25] Zohar AH, Denollet J, Lev Ari L, Cloninger CR (2011). The psychometric properties of the DS14 in Hebrew and the prevalence of type D personality in Israeli adults. Eur J Psychol Assess.

[26] Sprösser G, Klusmann V, Ruby MB, Arbit N, Rozin P, Schupp HT (2018). The positive eating scale: relationship with objective health parameters and validity in Germany, the USA and India. Psychol Health.

[27] Bentler PM, Bonett DG (1980). Significance tests and goodness of fit in the analysis of covariance structures. Psychol Bull.

[28] Browne MW, Cudeck R, Bollen KA, Long JS (1993). Alternative ways of assessing model fit. Testing structural equation models.

[29] Hilbert A, De Zwaan M, Braehler E (2012). How frequent are eating disturbances in the population? Norms of the eating disorder examination-questionnaire. PLoS One.

